# Differentiation of Gastric *Helicobacter* Species Using MALDI-TOF Mass Spectrometry

**DOI:** 10.3390/pathogens10030366

**Published:** 2021-03-18

**Authors:** Helena Berlamont, Chloë De Witte, Sofie De Bruyckere, James G. Fox, Steffen Backert, Annemieke Smet, Filip Boyen, Freddy Haesebrouck

**Affiliations:** 1 Department of Pathology, Bacteriology and Poultry Diseases, Faculty of Veterinary Medicine, Ghent University, 9820 Merelbeke, Belgium; Chloe.DeWitte@UGent.be (C.D.W.); Sofie.DeBruyckere@UGent.be (S.D.B.); 2 Division of Comparative Medicine, Massachusetts Institute of Technology, Cambridge, MA 02139, USA; jgfox@mit.edu; 3 Department Biology, Division Microbiology, University Erlangen Nuremberg, 91058 Erlangen, Germany; steffen.backert@fau.de; 4 Laboratory of Experimental Medicine and Pediatrics, Faculty of Medicine and Health Sciences, University of Antwerp, 2610 Antwerp, Belgium; Annemieke.Smet@uantwerpen.be

**Keywords:** gastric *Helicobacter* species, NHPH, MALDI-TOF MS

## Abstract

Gastric helicobacters (*Helicobacter* (*H.*) *pylori* and non-*H. pylori Helicobacter* species (NHPHs)) colonize the stomach of humans and/or animals. *Helicobacter* species identification is essential since many of them are recognized as human and/or animal pathogens. Currently, *Helicobacter* species can only be differentiated using molecular methods. Differentiation between NHPHs using MALDI-TOF MS has not been described before, probably because these species are poorly represented in current MALDI-TOF MS databases. Therefore, we identified 93 gastric *Helicobacter* isolates of 10 different *Helicobacter* species using MALDI-TOF MS in order to establish a more elaborate *Helicobacter* reference database. While the MALDI Biotyper database was not able to correctly identify any of the isolates, the in-house database correctly identified all individual mass spectra and resulted in 82% correct species identification based on the two highest log score matches (with log scores ≥2). In addition, a dendrogram was constructed using all newly created main spectrum profiles. Nine main clusters were formed, with some phylogenetically closely related *Helicobacter* species clustering closely together and well-defined subclusters being observed in specific species. Current results suggest that MALDI-TOF MS allows rapid differentiation between gastric *Helicobacter* species, provided that an extensive database is at hand and variation due to growth conditions and agar-medium-related peaks are taken into account.

## 1. Introduction

Helicobacters are Gram-negative bacteria that naturally colonize the gastrointestinal tract of humans and various animal species [1,2]. The *Helicobacter* genus can roughly be divided into two major groups: gastric species colonizing the stomach and enterohepatic species colonizing the intestinal tract and/or liver of their host [3,4,5,6]. *Helicobacter* (*H*.) *pylori* is the best studied and most prevalent *Helicobacter* species colonizing the human stomach, with an estimated worldwide prevalence of 44.3% [7]. Infections with this microorganism have been associated with gastritis, peptic ulcer disease, mucosa-associated lymphoid tissue (MALT-) lymphoma and gastric adenocarcinoma [8]. Gastric disease in humans has also been associated with other, spiral-shaped non-*H. pylori Helicobacter* species (NHPHs) naturally colonizing the stomach of various animals [2,9]. So far, detected zoonotic NHPHs are *H. suis* from pigs, *H. felis*, *H. bizzozeronii*, *H. salomonis*, and *H. heilmannii* from cats and dogs, and possibly *H. cetorum* from marine mammals, and “*Candidatus H. bovis*” from cattle [2,10,11,12,13]. The prevalence of NHPHs in human patients with gastric complaints is estimated to range from 0.2% to 6% [2]. Nevertheless, due to difficulties in the diagnosis of NHPH infections, these numbers are most probably an underestimation of their true prevalence. Other animal-associated gastric *Helicobacter* species, for which a zoonotic potential has not yet been described, are *H. baculiformis* and *H. ailurogastricus* (cats), *H. cynogastricus* (dogs), *H. mustelae* (ferrets), *H. suncus* (house musk shrews), *H. acinonychis* (wild large felines), *H. labacensis*, *H. mehlei*, and *H. vulpis* (red foxes) and “*Candidatus H. homininae”* (gorilla, chimpanzee) [13,14,15,16]. Furthermore, the zoonotic potential of *H. suis* isolated from non-human primates has not been described so far. Animals can be subclinically infected with these gastric *Helicobacter* species or may develop gastric pathologies [2].

Identification to the species level of *Helicobacter* infections has become increasingly important, since many of them are recognized as important human and/or animal pathogens [2,13]. Correct species identification is also important to better estimate the true prevalence of NHPH infections in humans, to allow identification of the source of infection (e.g., in pets), and hence, allow implementation of correct measurements to avoid reinfection (e.g., treatment of infected pets). Finally, as antimicrobial resistance in NHPHs has already been described and since antimicrobial susceptibility can differ between *Helicobacter* species, species identification may guide antimicrobial therapy as well [17,18]. So far, however, no ideal diagnostic tool exists to identify *Helicobacter* infections to the species level. The feasibility of biochemical methods for *Helicobacter* identification and differentiation has been hampered because of the low metabolic activity of these bacteria and the limited number of available test characteristics [2,19]. Although the spiral-shaped NHPHs may be distinguished from the curve-shaped *H. pylori* by high-resolution microscopy, further species differentiation based on morphology is not reliable due to overlapping phenotypic characteristics [20]. Nowadays, *Helicobacter* species can only be differentiated using molecular methods, such as (q)PCR and sequencing of the *16S rRNA*, *ureAB*, *gyrB* and/or *hsp60* genes [21]. These diagnostic tools, however, are labor-intensive, time-consuming, and expensive.

In recent years, matrix-assisted laser desorption/ionization–time-of-flight–mass spectrometry (MALDI-TOF MS) has been introduced as an accurate, rapid, and inexpensive tool for microbial identification and diagnosis [22,23,24]. This technique is based on the generation of complex fingerprints of specific biomarker molecules by measuring the exact mass/charge ratio of peptides and proteins and might be an alternative method to identify and differentiate *Helicobacter* species [25]. MALDI-TOF MS is also increasingly used in polyphasic taxonomic approaches when describing new bacterial species [26]. Recently, three novel *Helicobacter* species (i.e., *H. labacensis*, *H. mehlei*, and *H. vulpis*) from the gastric mucosa of red foxes were described using this approach [16]. Inadequate MALDI-TOF MS identification may occur due to the incompleteness of mass spectrometry databases [27]. For example, the current MALDI Biotyper reference database library (Bruker Daltonics, Bremen, Germany) only contains 24 *Helicobacter* entries, most of which are enterohepatic *Helicobacter* species (i.e., *H. canadensis* (1), *H. canis* (2), *H. cholecystus* (1), *H. cinaedi* (2), *H. fennelliae* (1), *H. pullorum* (9)) and only a few are gastric species (i.e., *H. mustelae* (1) and *H. pylori* (7)). In order to establish a more accurate *Helicobacter* species identification by MALDI-TOF MS, we generated main spectrum profiles from all available gastric *Helicobacter* species in our lab, resulting in an in-house *Helicobacter* database. The presence of a robust MALDI-TOF MS database, as provided by this research, is a prerequisite for future MALDI-TOF MS analysis on clinical samples, enabling rapid diagnosis of gastric *Helicobacter* infections.

## 2. Results

In general, MSPs belonging to the same isolate created at different timepoints generated high log scores (Table S1) and clustered closely together (Figures S1–S3), indicating good reproducibility of gastric *Helicobacter* MALDI-TOF MS spectra. Log differences between MSPs belonging to the same isolate ranged from 0.01 to 0.76, whereby log scores ≥2 were seen for all MSPs, except for one: *H. suis* HS5 MSP (Table S1).

In total, 93 isolates of 10 gastric *Helicobacter* species were analyzed using MALDI-TOF MS. Additionally, MSPs after both dry and biphasic cultivation were created for 23 isolates, resulting in an in-house *Helicobacter* database containing 116 MSPs. A representative peak spectrum for each gastric *Helicobacter* species used in this study is given in Figure S4. After completion of the in-house *Helicobacter* database, all individual spectra used to create each MSP (20–24 spectra per MSP) were matched to the MALDI Biotyper and in-house database. When comparing the 20–24 individual spectra with the MALDI Biotyper database, no logarithmic identification scores ≥1.70 were generated. When comparing the individual spectra with the newly created in-house database, correct species identification was seen for all isolates, with maximum log scores varying between 2.22 and 2.88 (Table 1). Log scores for the best incorrect species match varied between 1.96 and 2.61. When comparing the best incorrect match per species with the best correct match of that isolate, the log difference varied from 0.05 to 0.80 (Table 1). As expected, the majority (i.e., 97%) of the 20–24 individual spectra generated a first match with their own isolate’s MSP. Therefore, we also investigated whether correct species identification was seen for the second-best match (i.e., the isolate with the second highest log score). For the 20–24 individual spectra of 102 MSPs, the second-best match also belonged to the correct *Helicobacter* species, of which 95 had log scores ≥2. For the individual spectra of 14 MSPs, the second-best match did not belong to the correct *Helicobacter* species, with 8/14 (i.e., 3 *H. bizzozeronii*, 3 *H. felis*, 1 *H. cetorum*, and 1 *H. salomonis*) having a log score ≥2, 4/14 (i.e., 1 *H. ailurogastricus*, 1 *H. bizzozeronii*, 1 *H. cetorum*, and 1 *H. felis*) having a log score <2 and ≥1.70, and 2/14 (i.e., 1 *H. cetorum* and 1 *H. felis*) having a log score <1.70 (Table 2).

When comparing the individual spectra of the 23 isolate MSPs grown at biphasic conditions to the corresponding isolate MSP grown at dry conditions, and vice versa, log scores varied from <0 to 2.45 (Table 3). For 1 *H. baculiformis*, 1 *H. cynogastricus*, and 4 *H. felis* isolates, log scores were ≥2 for both comparisons. Conversely, for 1 *H. acinonychis*, 3 *H. felis,* and 1 *H. salomonis* isolate, log scores were <1.70 for both comparisons.

Additionally, unseeded culture media were analyzed and compared to the in-house *Helicobacter* database. Only brain–heart infusion (BHI) agar yielded MALDI-TOF MS peaks that matched to 1 *H. bizzozeronii*, 1 *H. cetorum*, 2 *H. felis,* and 1 *H. salomonis* isolates with log scores ≥2. Furthermore, log scores <2 and ≥1.70 were shown for 1 *H. bizzozeronii*, 2 *H. felis,* and 1 *H. salomonis* isolate (Table S2). Ten out of 12 peaks from BHI agar showed similar *m/z* [Da] values in *H. salomonis* Inkinen, *H. bizzozeronii* 12A, *H. cetorum* MIT 01-6096, *H. felis* M38, and/or *H. felis* M42 MSP (Table S3). All isolates showing similar peaks to those of BHI agar were grown at dry conditions using BHI agar.

**Figure 1:**
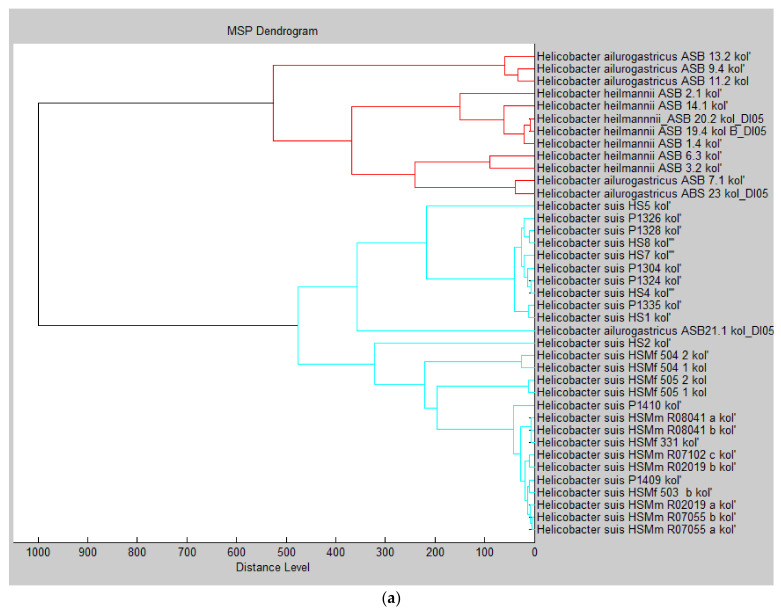

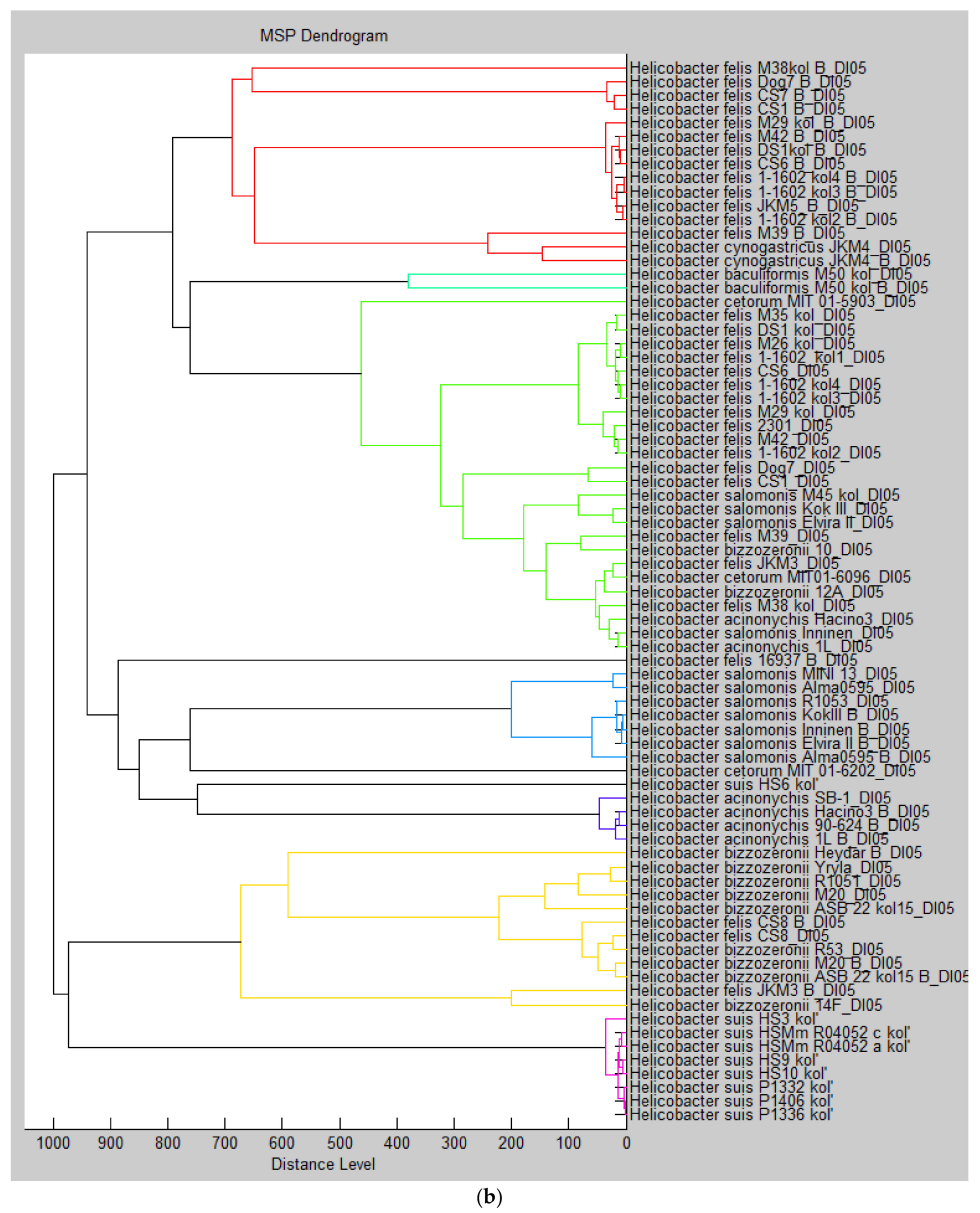
Dendrogram of 116 main spectrum profiles (MSPs) from gastric *Helicobacter* isolates used to create the in-house *Helicobacter* database. The dendrogram created with MBT Compass Explorer 4.1 (Bruker Daltonics) of 116 *Helicobacter* isolate MSPs showed 9 main clusters, with (**a**) 1 *H. suis* cluster and 1 cluster with *H. heilmannii* and *H. ailurogastricus* and (**b**) a second *H. suis* cluster, 1 cluster with *H. felis* and *H. cynogastricus;* 1 *H. baculiformis* cluster; 1 cluster containing *H. felis, H. cetorum*, some *H. salomonis*, *H. bizzozeronii,* and *H. acinonychis* isolates; 1 *H. salomonis* cluster; 1 *H. acinonychis* cluster; and 1 *H. bizzozeronii* cluster with some *H. felis* isolates. All *H. ailurogastricus* (except *H. ailurogastricus* ASB 21.1), *H. heilmannii,* and *H. suis* isolates were grown under biphasic culture conditions on *Brucella* agar + *Brucella* broth. *H. ailurogastricus* ASB 21.1 was grown under dry culture conditions on *Brucella* agar. All *H. acinonychis*, *H. baculiformis*, *H. bizzozeronii*, *H. cetorum*, *H. cynogastricus*, *H. felis*, and *H. salomonis* isolates were grown under dry and/or biphasic (‘B’ mentioned behind isolate name) conditions on brain–heart infusion (BHI) agar ± BHI broth.

Species differentiation and diversity of spectra within a species were also assessed by constructing dendrograms based on a similarity matrix incorporated in the MBT Compass Explorer 4.1 software. Not all isolates within one species formed a cluster clearly distinct from other species (Figure 1a,b). Nine main clusters were generated: 2 *H. suis* clusters; 1 cluster with *H. heilmannii* and *H. ailurogastricus;* 1 cluster with *H. felis* and *H. cynogastricus;* 1 *H. baculiformis* cluster; 1 cluster containing *H. felis*, *H. cetorum*, and some *H. salomonis*, *H. bizzozeronii,* and *H. acinonychis* isolates; 1 *H. salomonis* cluster; 1 *H. acinonychis* cluster; and 1 *H. bizzozeronii* cluster with some *H. felis* isolates. Additionally, 1 *H. felis* isolate (16937), 1 *H. cetorum* isolate (MIT 01-6202), and 1 *H. suis* isolate (HS6) were found separated from the main clusters. Remarkably, one *H. ailurogastricus* isolate (ASB 21.1) clustered in one of the *H. suis* clusters. Except for *H. ailurogastricus* ASB 21.1, all *H. ailurogastricus* and *H. heilmannii* isolates clustered together, with *H. ailurogastricus* ASB 7.1 and ASB 23 clustering closer to *H. heilmannii* than to other *H. ailurogastricus* isolates. Although *H. felis* and *H. cynogastricus* clustered together, *H. cynogastricus* was situated rather distinctly from most *H. felis* isolates, except from the biphasically cultivated *H. felis* M39. Most *H. bizzozeronii* isolates clustered together, except for 2 isolates (10 and 12A), which clustered in one of the *H. felis* groups. Four *H. salomonis* isolates (M45, Kok III dry, Elvira II dry, and Inkinen dry) clustered in one of the *H. felis* groups, whereas isolates Kok III, Elvira II, and Inkinen after biphasic cultivation did belong to the main *H. salomonis* cluster. Similarly, two *H. acinonychis* isolates (Hacino3 dry and 1L dry) clustered in one of the *H. felis* groups, whereas both isolates grown at biphasic conditions did belong to the main *H. acinonychis* cluster.

## 3. Discussion

In order to accurately identify bacterial strains using MALDI-TOF MS, a complete and representative database should be present [28]. As the current commercial MALDI Biotyper database is far from complete for *Helicobacter* species, we aimed at extending it with 93 isolates of 10 different gastric *Helicobacter* species to facilitate future MALDI-TOF MS identification. While most gastric *Helicobacter* species were included, there are still some species lacking, such as *H. suncus*, “*Candidatus H. bovis*”, and “*Candidatus H. hominae*”. Furthermore, as the number of isolates available for some species is currently limited, additional isolation and completion of the database is warranted. However, obtaining additional isolates is not evident due to the fastidious nature of these *Helicobacter* species [2]. Indeed, the collection of isolates used in this study was a process that took almost 20 years [29]. Therefore, validation of the newly created in-house *Helicobacter* database was performed with isolates available in our lab. It was decided not to include *H. mustelae* in our study as this species is phylogenetically more related to enterohepatic *Helicobacter* species than to the gastric ones [29,30,31].

Although the reproducibility of the created spectra in our study was shown to be good, some isolate MSPs deviated from the other MSPs of the same isolate. This divergence is most likely due to spotting of suboptimal bacterial concentrations, further emphasizing the need of sufficient and standardized bacterial concentrations for MALDI-TOF MS identification, especially for fastidiously growing bacterial species [32,33].

According to Bruker recommendations, no reliable identification at genus level (log score ≥1.70) or species level (log score ≥2) was possible with the most recent MALDI Biotyper database. However, after completion of the in-house *Helicobacter* database, correct species identification was seen for 100% of the individual mass spectra. Correct species identification based on the second-best match was observed for 82% (95/116) of the individual mass spectra with log scores ≥2 and 6% (7/116) of the individual mass spectra with log scores <2, whereas incorrect species identification occurred for 7% (8/116) of the individual mass spectra with log scores ≥2 and for 5% (6/116) with log scores <2. The dendrogram also showed separate clustering of most (i.e., 9/14) of these latter incorrectly identified isolates from other isolates of that species. This indicates that variation between isolates of the same *Helicobacter* species might occur, underlining the need to establish an elaborate reference database containing as many isolates as possible. Hence, correct *Helicobacter* species identification, even with more aberrant isolates, should be possible. Overall, MALDI-TOF MS may correctly identify most *Helicobacter* species, although caution remains warranted.

While MALDI-TOF MS is considered a rapid and accurate tool for microbial identification, certain drawbacks must be considered [28,34]. One of the major limitations is that bacteria must be cultured prior to analysis, which decelerates the identification process. Indeed, due to their fastidious nature, gastric *Helicobacter* species are extremely difficult to isolate and cultivate *in vitro* [2,8]. The time needed for cultivation depends on the *Helicobacter* species and can vary between 24 and 72 h. Nevertheless, whenever an isolate is at hand, identification can be obtained within 10–30 min. While MALDI-TOF MS identification protocols without any or only limited prior cultivation have already been developed for body fluids such as blood; urine; and peritoneal, synovial, broncho-alveolar lavage; and cerebrospinal fluid [35,36,37,38], this is not the case for stomach fluids and mucosa. Development of a direct protocol is most likely hampered due to the difficulty of obtaining pure bacterial samples with sufficient concentrations (10^6^–10^8^ CFU/mL for certain fastidiously growing bacteria) and due to the presence of host (human/animal) material contamination, such as mucus proteins [33,39,40]. Nevertheless, development of a direct MALDI-TOF MS identification protocol is still of interest, as it would allow fast diagnosis in clinical practice, thereby providing more accurate prevalence data concerning gastric NHPH infections in human patients and animal hosts. The development of a protocol enabling MALDI-TOF MS identification on clinical gastric samples, even with prior cultivation, will take several months or even years and requires several critical steps, i.e., the search of different animal patients suspected to carry gastric *Helicobacter* spp., the collection of stomach samples of these animals (either alive or when recently died), and, most importantly, the optimization of culture conditions enabling growth of the bacteria in a liquid phase and simultaneously suppressing the growth of contaminants.

Another drawback is that culture conditions might affect the microbial physiology and protein expression profile of bacteria, causing alterations in the MALDI-TOF MS fingerprint [41]. The impact of cultivation medium on the quality (i.e., intensity and number of peaks) of MALDI-TOF MS spectra has already been reported for different bacterial species, including enterohepatic *Helicobacter* species [42,43,44,45,46]. Indeed, in our study, great spectral differences were revealed for 17 of 23 isolates cultured under both dry and biphasic conditions, while similar spectra were observed for the other 6 isolates (Table 3). Despite the impact on MSPs, the second-best match of the majority of the isolates cultured dry and biphasically did belong to the correct *Helicobacter* species. This was, however, not the case for several dry-cultured *H. felis* isolates and one dry-cultured *H. salomonis* isolate. In our study, 2 different types of medium (i.e., *Brucella* agar ± broth and BHI agar ± broth) were used for cultivation of gastric *Helicobacter* isolates. Only BHI agar generated MALDI-TOF peaks, of which the majority could also be demonstrated in the peak spectra of some *Helicobacter* isolates grown under dry conditions using BHI agar (Table S2). The earlier mentioned incorrect second-best match identifications of dry-cultured *H. felis* and *H. salomonis* isolates could thus be attributed to overlap in medium-specific peaks with other dry-cultured *Helicobacter* isolates. Indeed, the BHI agar-related peaks resulted in false-positive *H. bizzozeronii*, *H. cetorum*, *H. felis* and *H. salomonis* identifications. Recently, this phenomenon has also been described for MALDI-TOF MS identification of certain *Mycoplasma* species [47,48]. Gastric *Helicobacter* MALDI-TOF MS spectra of isolates grown under dry conditions using BHI agar might thus be contaminated with irrelevant medium-specific peaks, which might lead to incorrect identifications and great spectral differences between isolates grown under dry or biphasic conditions. Therefore, contamination with agar should be avoided as much as possible when spotting the bacteria onto the MALDI-TOF target plate, and, if feasible, cultivation under biphasic conditions should be considered.

Cultivation duration has also been shown to affect MALDI-TOF MS spectrum quality [25,44,49]. In general, it is advised to use fresh colonies for analysis (i.e., less than 48 h old) as spectra become weaker with increasing cultivation time, most likely due to ribosomal protein degradation [25]. However, cultivation length is mainly species dependent and for some bacteria, such as *Listeria* species, it has already been shown that extended periods of growth do not affect MALDI-TOF MS spectrum quality [50]. Because of the relatively slow growth and formation of small colonies, all gastric *Helicobacter* isolates were grown for 48–72 h to ensure sufficient material for MALDI-TOF MS identification. The yielded spectra were shown to be good, even after 72 h of incubation, further indicating that longer cultivation times can be feasible for MALDI-TOF analyses in certain bacterial species.

Unlike the phylogenetic tree based on the core genome of gastric *Helicobacter* species (Figure S5), no strict clustering of the different species was seen in the MALDI-TOF MS dendrogram. While two separate *H. suis* clusters were visible, both contained mixed isolates from pigs and from non-human primates, indicating that the host species of origin does not play a major role in the formation of the MALDI-TOF subclusters. Furthermore, *H. suis* HS6 did not belong to either cluster, although *H. suis* HS1 to HS10 were all isolated from the gastric mucosa of sows using the same method [51,52]. The reason for the separate *H. suis* clusters remains, thus, unclear. The clustering of *H. ailurogastricus* with *H. heilmannii* is not a surprise as both species are phylogenetically very similar [53]. Subtyping within *H. ailurogastricus* has already been proposed, with *H. ailurogastricus* ASB 7.1, 21.1, and 23 showing more DNA overlap with *H. heilmannii* compared to *H. ailurogastricus* ASB 9.4, 11.2, and 13.2 [29,53]. This could thus explain the closer clustering of *H. ailurogastricus* ASB 7.1 and 23 to *H. heilmannii* compared to the other *H. ailurogastricus* isolates. *H. ailurogastricus* ASB 21.1 was, unlike all other *H. ailurogastricus* isolates, grown under dry culture conditions, which might explain its distinct position in the dendrogram. The phylogenetically closely related *H. cynogastricus* and *H. felis* clustered together as well [29]. The clustering of some *H. salomonis*, *H. bizzozeronii,* and *H. acinonychis* isolates after dry cultivation within the main *H. felis* group could possibly be explained by the elimination of species-specific peaks when cultivated under dry conditions, leading to a peak pattern more similar to that of *H. felis*. Furthermore, the presence of medium-specific peaks might play an important role here as well, as discussed above. Similarly, *H. felis* JKM3 seems to lose *H. felis*–specific peaks when cultivated biphasically. The 3 *H. cetorum* isolates did not cluster together, which could possibly be explained by the difference in host species of isolation and/or living area of that host. MIT 01-5903 was isolated from a Pacific white-sided dolphin in Chicago (USA), whereas MIT 6096 and MIT 01-6202 originated an Atlantic bottle nose dolphin isolated in respectively Florida (USA) and Oahu (Hawaï) [54,55]. Finally, for some isolates, namely *H. felis* CS8 and 16937, there are no obvious reasons for the unusual localization in the dendrogram.

## 4. Materials and Methods

### 4.1. Helicobacter Isolates and Growth Conditions

To generate main spectrum profiles (MSPs), 35 *H. suis* isolates, 20 *H. felis* isolates, 9 *H. bizzozeronii* isolates, 7 *H. salomonis* isolates, 7 *H. heilmannii* isolates, 6 *H. ailurogastricus* isolates, 4 *H. acinonychis* isolates, 3 *H. cetorum* isolates, 1 *H. cynogastricus* isolate, and 1 *H. baculiformis* isolate were used. Genomes are available for 20 *H. suis* isolates, 19 *H. felis* isolates, 7 *H. bizzozeronii* isolates, 7 *H. heilmannii* isolates, 6 *H. ailurogastricus* isolates, 5 *H. salomonis* isolates, 4 *H. acinonychis* isolates, 2 *H. cetorum* isolates, 1 *H. cynogastricus* isolate, and 1 *H. baculiformis* isolate used in our study. All isolates together with their culture conditions, host of isolation*, in vitro* passage, and genome accession number (if available) are listed in Table S4.

The *H. ailurogastricus*, *H. heilmannii*, and *H. suis* isolates were cultivated according to the method described earlier [17]. In brief, bacteria were grown on *Brucella* agar (Becton-Dickinson, Erembodegem, Belgium) supplemented with 20% inactivated fetal calf serum (Hyclone, Thermo Fisher Scientific, Waltham, MA, USA), 5 mg/L amphotericin B (Sigma-Aldrich, Saint Louis, MO, USA), *Campylobacter* selective supplement (Skirrow, Oxoid, Basingstoke, UK; containing 10 mg/L vancomycin, 5 mg/L trimethoprim lactate, and 2500 U/L Polymyxin B), and Vitox supplement (Oxoid). For all *H. ailurogastricus* (except *H. ailurogastricus* ASB 21.1), *H. heilmannii*, and *H. suis* isolates, *Brucella* broth (Oxoid) was added on top of the agar to obtain biphasic culture conditions. The pH of both agar and broth was adjusted to 5 by adding HCl to a final concentration of 0.05%.

The *H. acinonychis*, *H. baculiformis*, *H. bizzozeronii*, *H. cetorum*, *H. cynogastricus*, *H. felis*, and *H. salomonis* isolates were grown on either brain–heart infusion (BHI) agar (Thermo Fisher Scientific, Waltham, MA, USA) supplemented with 10% defibrinated horse blood (bioTRADING, Mijdrecht, The Netherlands), or *Campylobacter* selective supplement (Skirrow, Oxoid, Basingstoke, UK; containing 10 mg/L vancomycin, 5 mg/L trimethoprim lactate, and 2500 U/L Polymyxin B), and Vitox supplement (Oxoid), or BHI broth (Thermo Fisher Scientific) was added on top of the agar to obtain biphasic culture conditions. Two *H. acinonychis* isolates, 1 *H. baculiformis* isolate, 2 *H. bizzozeronii* isolates, 1 *H. cynogastricus* isolate, 13 *H. felis* isolates, and 4 *H. salomonis* isolates were grown at both dry and biphasic conditions.

All isolates were grown for 48–72 h under microaerobic conditions (85% N_2_, 10% CO_2_, 5% O_2_) at 37 °C and were passaged at least twice to ensure reliable growth.

### 4.2. Sample Preparation for MALDI-TOF MS Analysis

Similar to the methods described earlier [39,48], a single colony of the dry cultures was removed from the agar plates using a toothpick, after which it was deposited on an MSP 384 target polished steel BC plate (Bruker Daltonics, Bremen, Germany) in 8 replicates and overlaid with 1 µL of α-cyano-4-hydroxycinnamic acid matrix solution (Bruker Daltonics, Bremen, Germany).

From the biphasic cultures, 500–700 µL was placed in 1.5 mL Eppendorf tubes and centrifugated at 2400× *g* for 5 min. Supernatants were decanted, and pellets were washed twice with 500 µL phosphate-buffered saline (PBS) and centrifugated for 5 min at 2400× *g*. Pellets were then resuspended in 20–100 µL PBS, after which 1 µL was spotted onto an MSP 384 target polished steel BC plate (Bruker Daltonics) in eight replicates, air dried and overlaid with 1 µL of α-cyano-4-hydroxycinnamic acid matrix solution (Bruker Daltonics).

No prior extraction protocol was performed as earlier tests revealed lower spectra quality (i.e., more variance and more dispersion of the peaks) compared to direct spotting.

### 4.3. Generation of (Reference) Spectra

Mass spectra were generated using Autoflex III smartbeam MALDI-TOF MS (Bruker Daltonics, Bremen, Germany) and flexControl 1.4 software, version 3.4 (Bruker Daltonics). A bacterial test standard (BTS, Bruker Daltonics) was used in each run for calibration purposes and as a quality control. To generate the MSPs of the different *Helicobacter* isolates, the method described by Spergser et al. [39] was used. In short, 24 individual mass spectra from each isolate were obtained, consisting of 3 replicate measurements of the 8 wells described earlier. The quality of raw spectra was then evaluated using flexAnalysis 3.4 software (Bruker Daltonics), whereby flatline spectra, spectra diverging from the cohort core, and spectra displaying high background noise were deleted. After smoothing and baseline subtraction, a minimum of 20 spectra of high quality were selected for MSP creation using standard settings of the automated MSP creation functionality in MBT Compass Explorer 4.1 (Bruker Daltonics). Resulting MSPs were consecutively added to the in-house *Helicobacter* project database using Compass Explorer software.

A dendrogram of all the in-house *Helicobacter* MSPs was then generated using MBT Compass Explorer 4.1. Using BIONUMERICS 7 software (Applied Maths), we tried to further investigate the MALDI-TOF MS peak spectra. The raw spectra were curated, and cluster analysis was performed (Figure S6). Although minor differences in clusters could be detected, in general, similar results as those obtained with flexAnalysis 3.4 and MBT Compass Explorer 4.1 software were acquired.

### 4.4. Testing of Reproducibility

To confirm the reproducibility of the generated MALDI-TOF MS spectra, several *H. ailurogastricus*, *H. heilmannii*, and *H. suis* isolates were kept in culture for a longer period of time (with passing them every 48–72 h) and were analyzed at different timepoints. New MSPs were then created and log scores as well as dendrograms of these gastric *Helicobacter* MSPs were subsequently produced (Table S1, Figures S1–S3).

### 4.5. Testing of Culture Conditions

To identify whether culture conditions influence *Helicobacter* MALDI-TOF MS peak spectra, MSPs of 23 isolates were created after both dry and biphasic cultivation. Log scores of corresponding spectra of the same isolate were recorded (Table 3). Additionally, unseeded culture media (*Brucella* agar, *Brucella* broth, BHI agar, and BHI broth) were analyzed by MALDI-TOF MS and resulting spectra were compared to those in the in-house *Helicobacter* database (Tables S2 and S3). Using the direct transfer method, a small amount (using a toothpick) of *Brucella* agar and BHI agar, and 1 µL of *Brucella* broth and BHI broth were spotted on a polished steel BC target plate (Bruker Daltonics) in 4 replicates. Spotted samples were covered with 1 µL of α-cyano-4-hydroxycinnamic acid matrix solution and processed as described earlier.

## 5. Conclusions

Even though gastric NHPHs are fastidiously growing organisms, MALDI-TOF MS allows rapid differentiation between gastric NHPH isolates, provided that an extensive database is at hand. In current research, a database consisting of 93 isolates representing 10 NHPHs was constructed. While correct species identification could not be achieved using the commercial database, the in-house database correctly identified all individual mass spectra and resulted in 82% correct species identification based on the two highest log score matches (with log scores ≥2). However, spectra obtained under different growing conditions, for example dry versus biphasic growing conditions, may differ, and agar-medium-related peaks may influence reliability of the obtained results, as observed for 4 NHPHs (i.e., *H. bizzozeronii*, *H. cetorum*, *H. felis*, and *H. salomonis*). Although current results suggest that subtyping below the species level might also be possible for certain species, such as *H. suis*, this should be confirmed in future investigations. The presence of a robust MALDI-TOF MS database, as provided by this research, is a prerequisite for future MALDI-TOF MS analysis on clinical samples. However, further research enabling (direct) MALDI-TOF MS identification on gastric samples is needed.

## Figures and Tables

**Table 1:** Validation of the in-house *Helicobacter* database with the 20–24 individual spectra of 116 main spectrum profiles (MSPs).

Species	Number of Isolates	Amount of MSPs Created	Hosts of Isolation	MALDI-TOF log Score Best Correct Match Range	Best Incorrect Match (Maximum log Score)	Log difference Best Correct Match − Best Incorrect Match
*H. acinonychis*	4	6	Wild felines	2.58–2.79	3rd match	0.12
					*H. salomonis* (2.42)	
*H. ailurogastricus*	6	6	Cat	2.49–2.82	7th match	0.80
					*H. heilmannii* (2.02)	
*H. baculiformis*	1	2	Cat	2.69–2.75	3rd match	0.67
					*H. cetorum* (2.02)	
*H. bizzozeronii*	9	11	Dog, cat, human	2.40–2.81	4th match *H. felis* (2.46)	0.08
*H. cetorum*	3	3	Dolphin	2.22–2.74	2nd match *H. bizzozeronii* (2.16)	0.06
*H. cynogastricus*	1	2	Dog	2.67–2.85	3rd match *H. felis* (2.23)	0.62
*H. felis*	20	33	Dog, cat	2.32–2.81	2nd match *H. bizzozeronii* (2.45)	0.14
*H. heilmannii*	7	7	Cat	2.56–2.82	6th match *H. ailurogastricus* (1.96)	0.64
*H. salomonis*	7	11	Dog	2.48–2.81	2nd match *H. acinonychis* (2.61)	0.05
*H. suis*	35	35	Pigs, non-human primates	2.45–2.88	5th match *H. felis* (2.08)	0.66
Total	93	116		2.22–2.88	1.96–2.61	0.05–0.80

**Table 2:** Individual spectra of isolate main spectrum profiles (MSPs) with second-best match not belonging to the correct *Helicobacter* species.

Individual Spectra of Isolate	Log Score Best MSP Match	Second-Best Match	Log Score Second-Best MSP Match *	Log Difference
*H. ailurogastricus* ASB 21.1 D	2.49	*H. heilmannii* ASB1.4 kol B	1.91	0.58
*H. bizzozeronii* 10 D	2.46	*H. felis* JKM3 D	2.34	0.12
*H. bizzozeronii* 12A D	2.40	*H. cetorum* MIT 01-6096 D	2.22	0.18
*H. bizzozeronii* Heydar B	2.78	*H. felis* CS8 B	1.87	0.91
*H. bizzozeronii* R1051 D	2.60	*H. felis* CS8 D	2.42	0.18
*H. cetorum* MIT 01-5903 D	2.74	*H. felis* M29 kol D	1.90	0.84
*H. cetorum* MIT 01-6096 D	2.22	*H. bizzozeronii* 12A D	2.16	0.06
*H. felis* CS8 D	2.59	*H. bizzozeronii* R1051 D	2.45	0.14
*H. felis* JKM3 D	2.57	*H. cetorum* MIT 01-6096 D	2.41	0.16
*H. felis* M38 D	2.45	*H. acinonychis* Hacino3 D	1.80	0.65
*H. felis* M39 D	2.54	*H. cetorum* MIT 01-6096 D	2.19	0.35
*H. salomonis* Inkinen D	2.66	*H. acinonychis* 1L D	2.61	0.05

* Second-best MSP matches with log scores <1.70 are not listed; D: dry cultivation; B: biphasic cultivation.

**Table 3:** Logarithmic identification scores of isolates at different culture conditions.

Species	Isolate	Log Score B to D	Log Score D to B
*H. acinonychis*	1L	0.76	1.48
	Hacino3	1.10	1.79
*H. baculiformis*	M50	2.45	2.44
*H. bizzozeronii*	M20	1.97	2.13
	ASB 22 kol 15	1.72	2.16
*H. cynogastricus*	JKM4	2.34	2.43
*H. felis*	CS1	2.22	2.06
	CS6	1.83	1.65
	CS8	2.31	1.92
	DS1	2.12	2.30
	Dog7	1.97	1.97
	JKM3	1.67	1.02
	M29	2.09	2.31
	M38	1.76	1.31
	M39	< 0	1.04
	M42	1.56	1.66
	1-1602 kol2	1.78	1.30
	1-1602 kol3	2.05	2.00
	1-1602 kol4	2.16	1.75
*H. salomonis*	KokIII	1.90	1.56
	Alma0595	2.10	1.51
	Inkinen	0.69	<0
	Elvira II	1.98	1.90
		<0–2.45	<0–2.44

Log score B to D: correspondence of the 20–24 individual spectra of the isolate grown at biphasic conditions compared to the isolate main spectrum profile (MSP) grown at dry conditions. Log score D to B: correspondence of the 20–24 individual spectra of the isolate grown at dry conditions compared to the isolate MSP grown at biphasic conditions. Green log score values: ≥2 acceptable for identification at species level; orange log score values: ≥1.70 acceptable for identification at genus level; red log score values <1.70 (Bruker recommendations).

## Data Availability

The data presented in this study are available in the article and the supplemental material.

## Supplementary Materials

The following are available online at https://www.mdpi.com/2076-0817/10/3/366/s1, Figure S1: Dendrogram of H. ailurogastricus isolates identified with MALDI-TOF MS at different timepoints, Figure S2: Dendrogram of H. heilmannii isolates identified with MALDI-TOF MS at different timepoints, Figure S3: Dendrogram of H. suis isolates identified with MALDI-TOF MS at different timepoints, Figure S4: A representative peak spectrum for each gastric Helicobacter species included in this study, Figure S5: Phylogenetic tree based on available core genomes of gastric Helicobacter isolates included in the MALDI-TOF MS analyses, Figure S6: BioNumerics dendrogram of 116 main spectrum profiles (MSPs) from gastric Helicobacter isolates used to create the in-house Helicobacter database, Table S1: Log scores of isolates with main spectrum profiles (MSPs) created at different timepoints, Table S2: Logarithmic identification score matches of brain–heart infusion (BHI) agar with the in-house Helicobacter database, Table S3: Overview of spectra (m/z)[Da] showing peaks of brain–heart infusion (BHI) agar similar to H. salomonis Inkinen, H. bizzozeronii 12A, H. cetorum MIT 01-6069, and H. felis M38 and M42 (within a deviation range of 10 Da), Table S4: Helicobacter isolates used in the current study to construct the in-house Helicobacter main spectrum profile (MSP) database.
