# Supplementary material for: Differentiation of Gastric Helicobacter Species Using MALDI-TOF Mass Spectrometry
Source: Pathogens. 2021 Mar 18;10(3):366. doi: 10.3390/pathogens10030366 (PMC8003121; doi:10.3390/pathogens10030366)
Supplement: Supplementary file 1 [file pathogens-10-00366-s001.zip › Figure S3.docx]

**Figure S3.** Dendrogram of *H. suis* isolates identified with MALDI-TOF MS at different timepoints


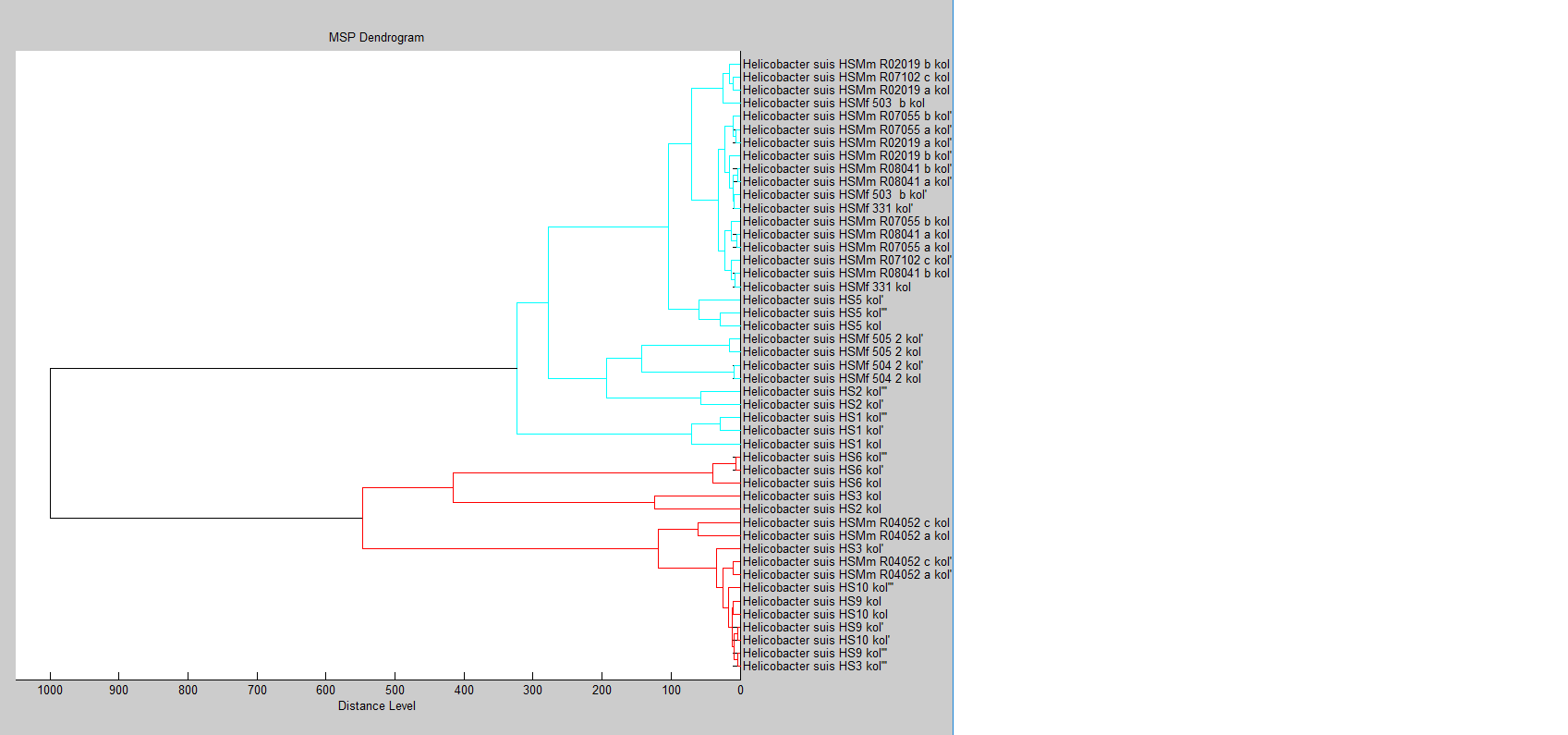


The dendrogram was created with MBT Compass Explorer 4.1 (Bruker Daltonics).

All *H. suis* isolates were grown under biphasic culture conditions on *Brucella* agar + *Brucella* broth.
