# Supplementary material for: Differentiation of Gastric Helicobacter Species Using MALDI-TOF Mass Spectrometry
Source: Pathogens. 2021 Mar 18;10(3):366. doi: 10.3390/pathogens10030366 (PMC8003121; doi:10.3390/pathogens10030366)
Supplement: Supplementary file 1 [file pathogens-10-00366-s001.zip › Figure S4.docx]

**Figure S4.** A representative peak spectrum for each gastric *Helicobacter* species included in this study

*H. acinonychis H. ailurogastricus*

**
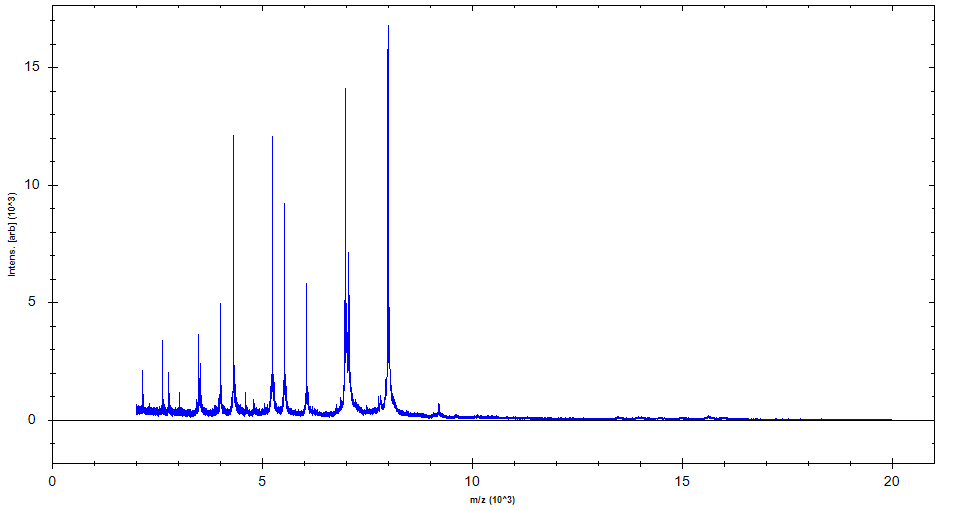

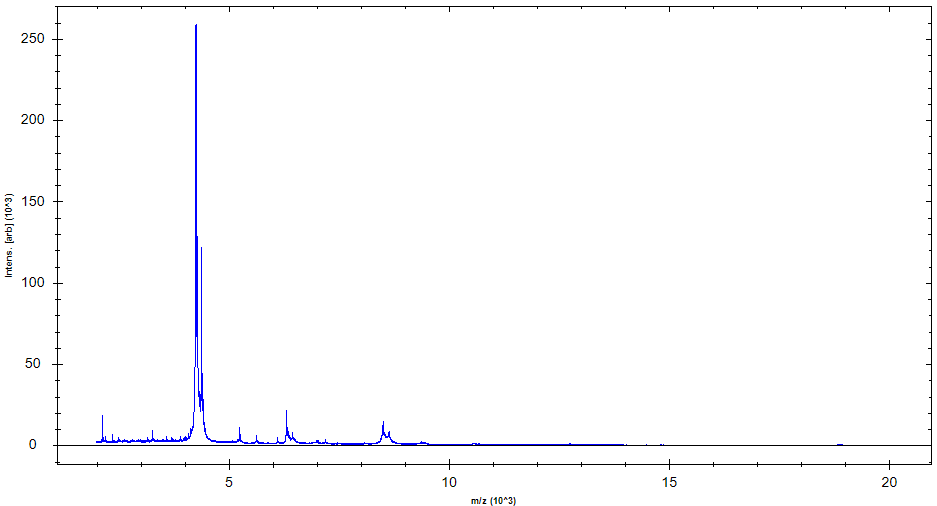
**

*H. baculiformis H. bizzozeronii*

**
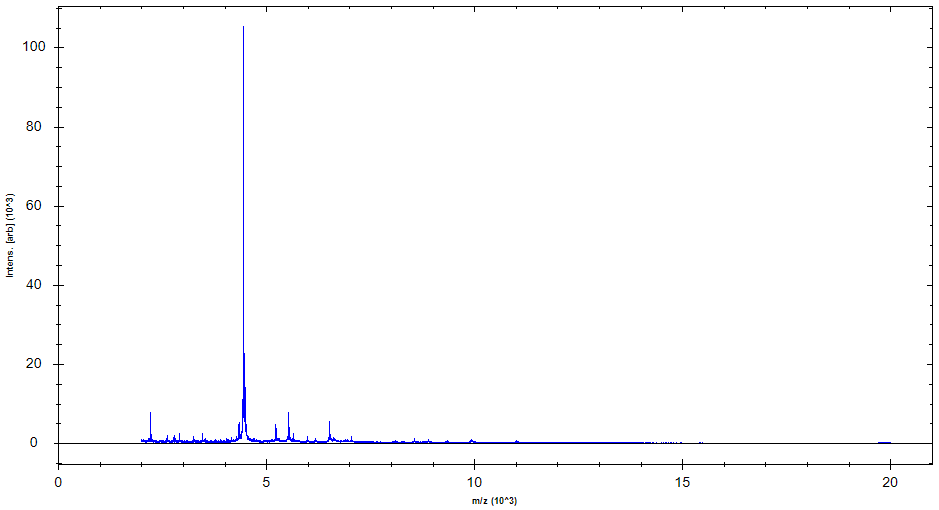

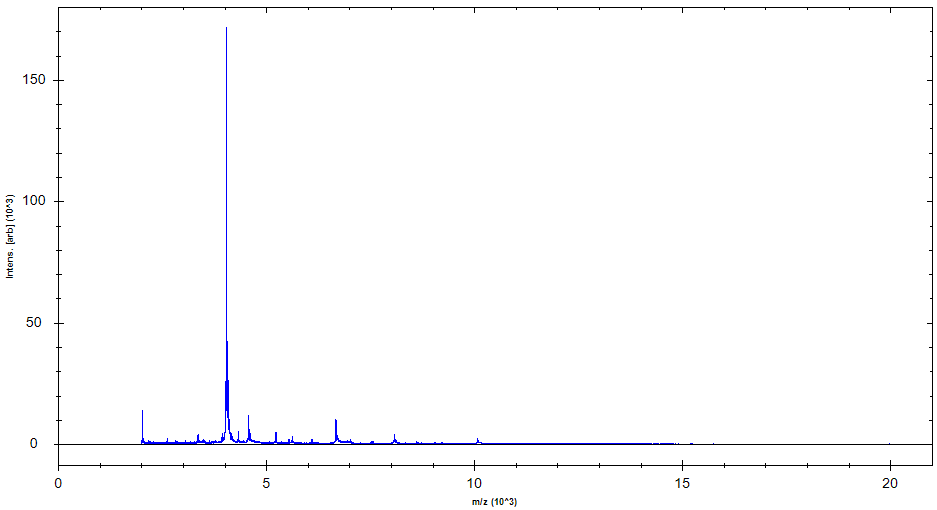
**

*H. cetorum H. cynogastricus*

*
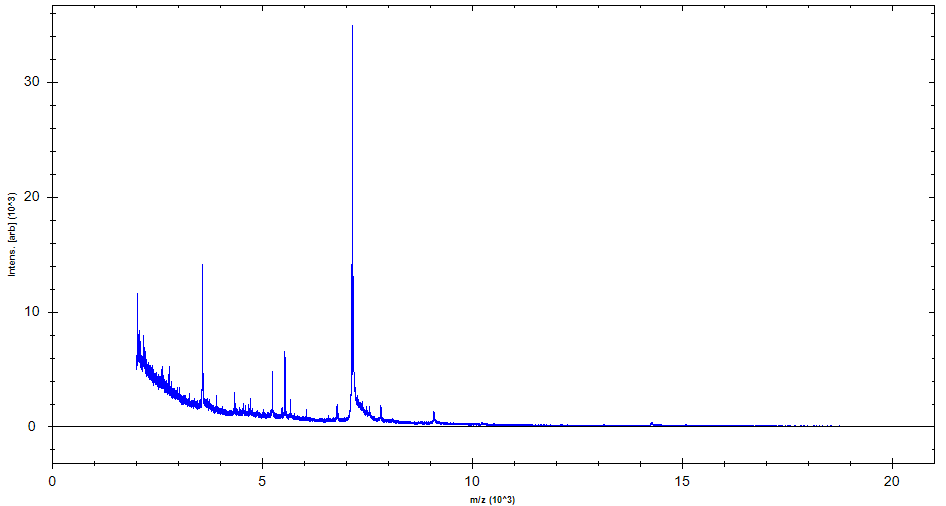

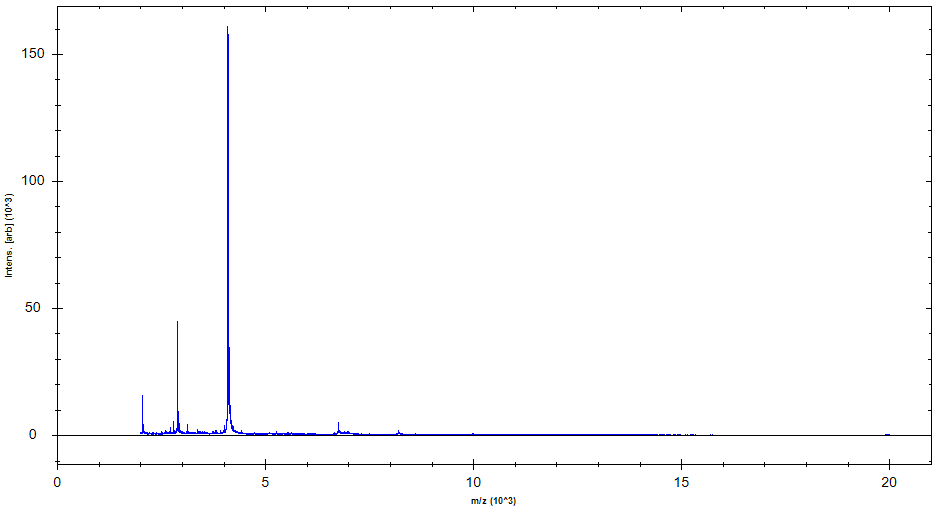
*

*H. felis H. heilmannii*

*
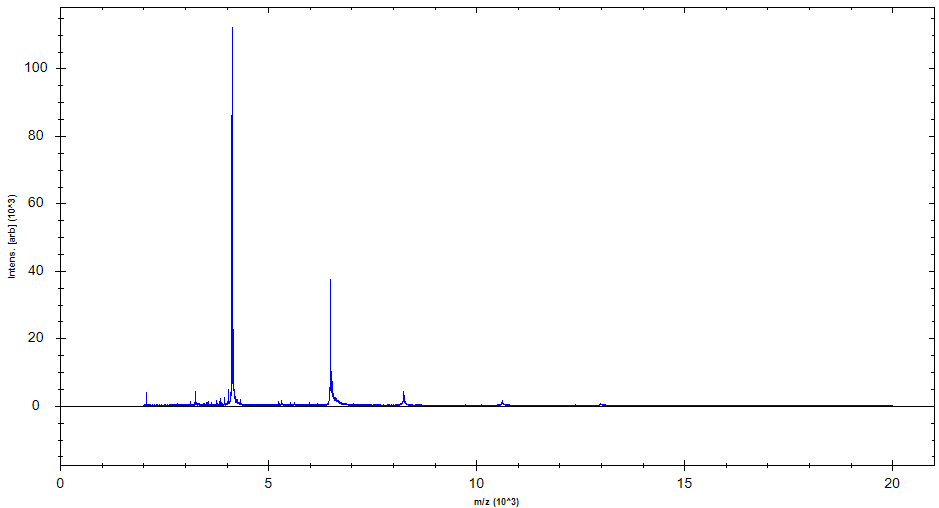

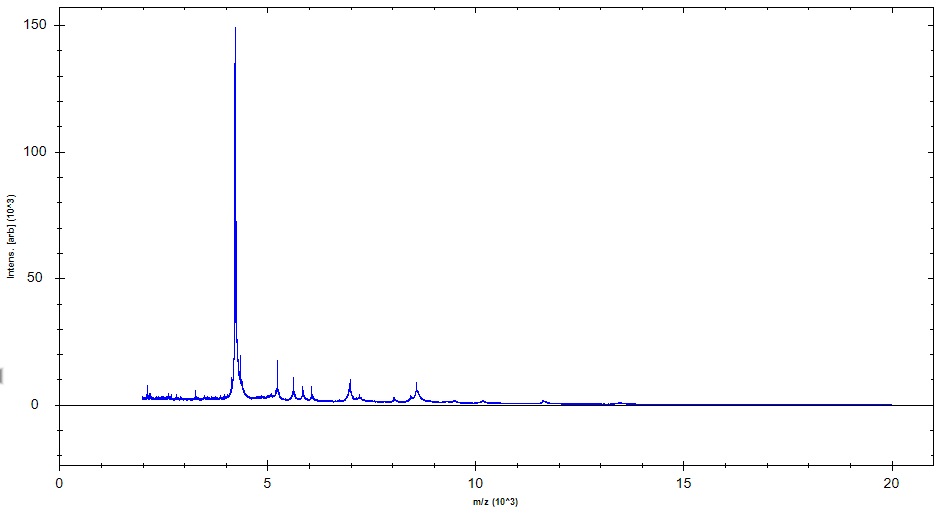
*

*H. salomonis H. suis*

*
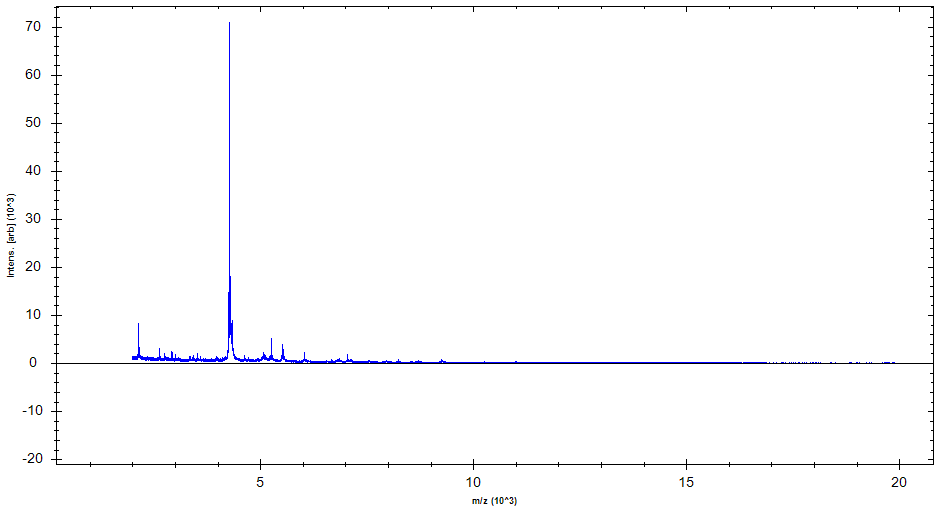

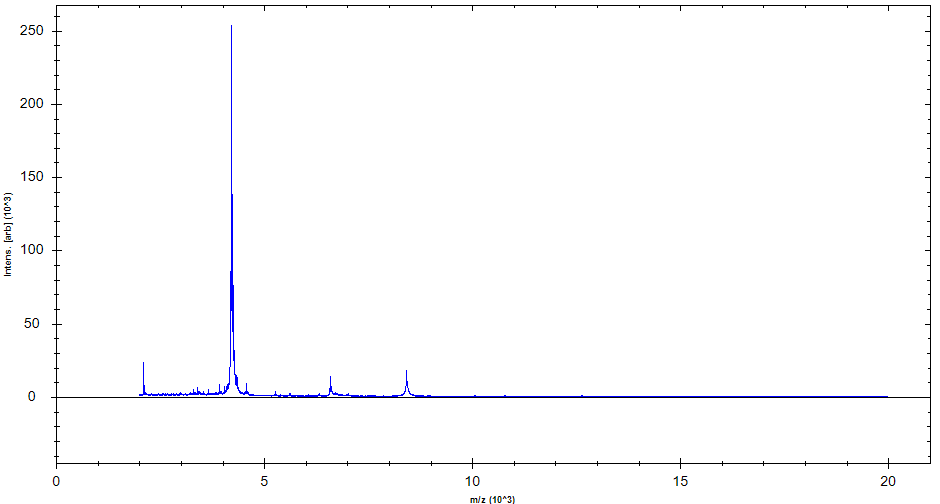
*

All peaks were extracted from the MBT Compass Explorer 4.1 software (Bruker Daltonics).
