# Supplementary material for: Differentiation of Gastric Helicobacter Species Using MALDI-TOF Mass Spectrometry
Source: Pathogens. 2021 Mar 18;10(3):366. doi: 10.3390/pathogens10030366 (PMC8003121; doi:10.3390/pathogens10030366)
Supplement: Supplementary file 1 [file pathogens-10-00366-s001.zip › Figure S5.docx]

**Figure S5.** Phylogenetic tree based on available core genomes of gastric *Helicobacter* isolates included in the MALDI-TOF MS analyses


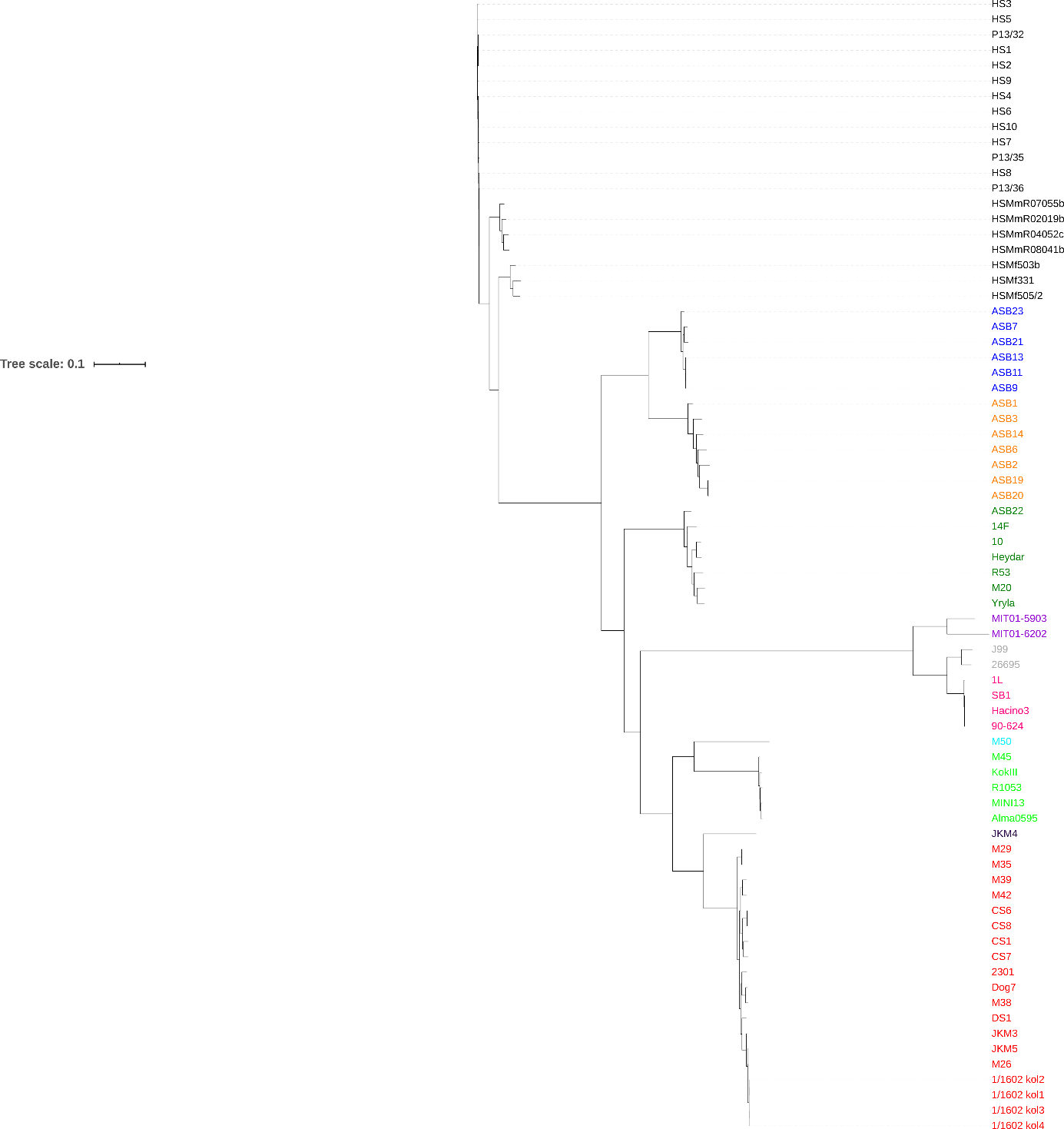


*H. suis* isolates (black); *H. ailurogastricus* isolates (blue); *H. heilmannii* isolates (orange); *H. bizzozeronii* isolates (khaki); *H. cetorum* isolates (purple); *H. pylori* isolates (grey); *H. acinonychis* isolates (magenta); *H. baculiformis* isolate (cyan); *H. salomonis* isolates (fluo green); *H. cynogastricus* isolate (dark purple); *H. felis* isolates (red).

The phylogenetic tree was built using the randomized accelerated maximum likelihood (RAxML) program by applying the -f a, -p 12345, -x 12345, -# 100, -m GTRGAMMA parameters, and visualized using the interactive tree of life (iTOL) software.
