# Supplementary material for: Differentiation of Gastric Helicobacter Species Using MALDI-TOF Mass Spectrometry
Source: Pathogens. 2021 Mar 18;10(3):366. doi: 10.3390/pathogens10030366 (PMC8003121; doi:10.3390/pathogens10030366)
Supplement: Supplementary file 1 [file pathogens-10-00366-s001.zip › Figure S6.docx]

**Figure S6.** BIONUMERICS dendrogram of 116 main spectrum profiles (MSPs) from gastric *Helicobacter* isolates used to create the in-house *Helicobacter* database


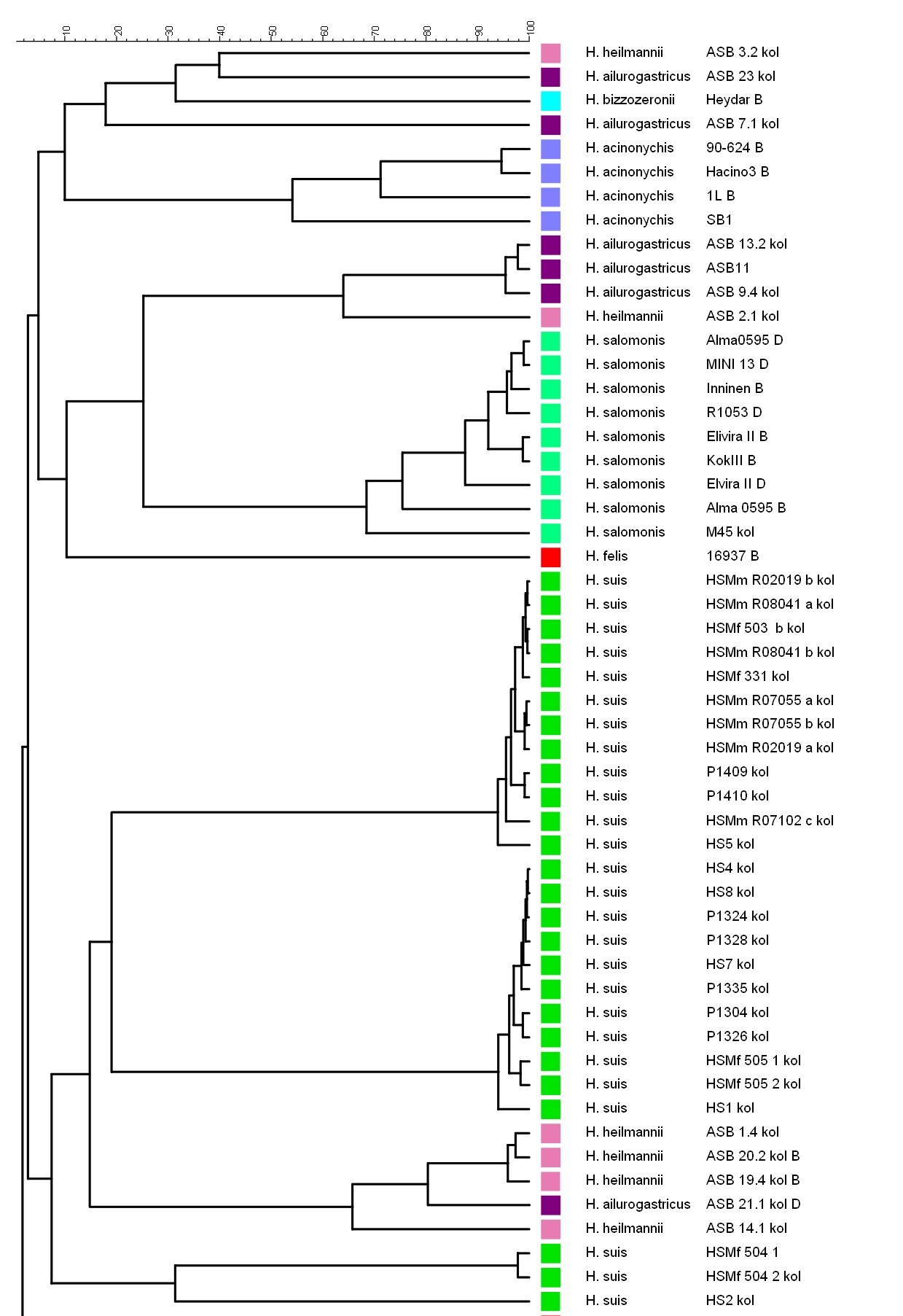


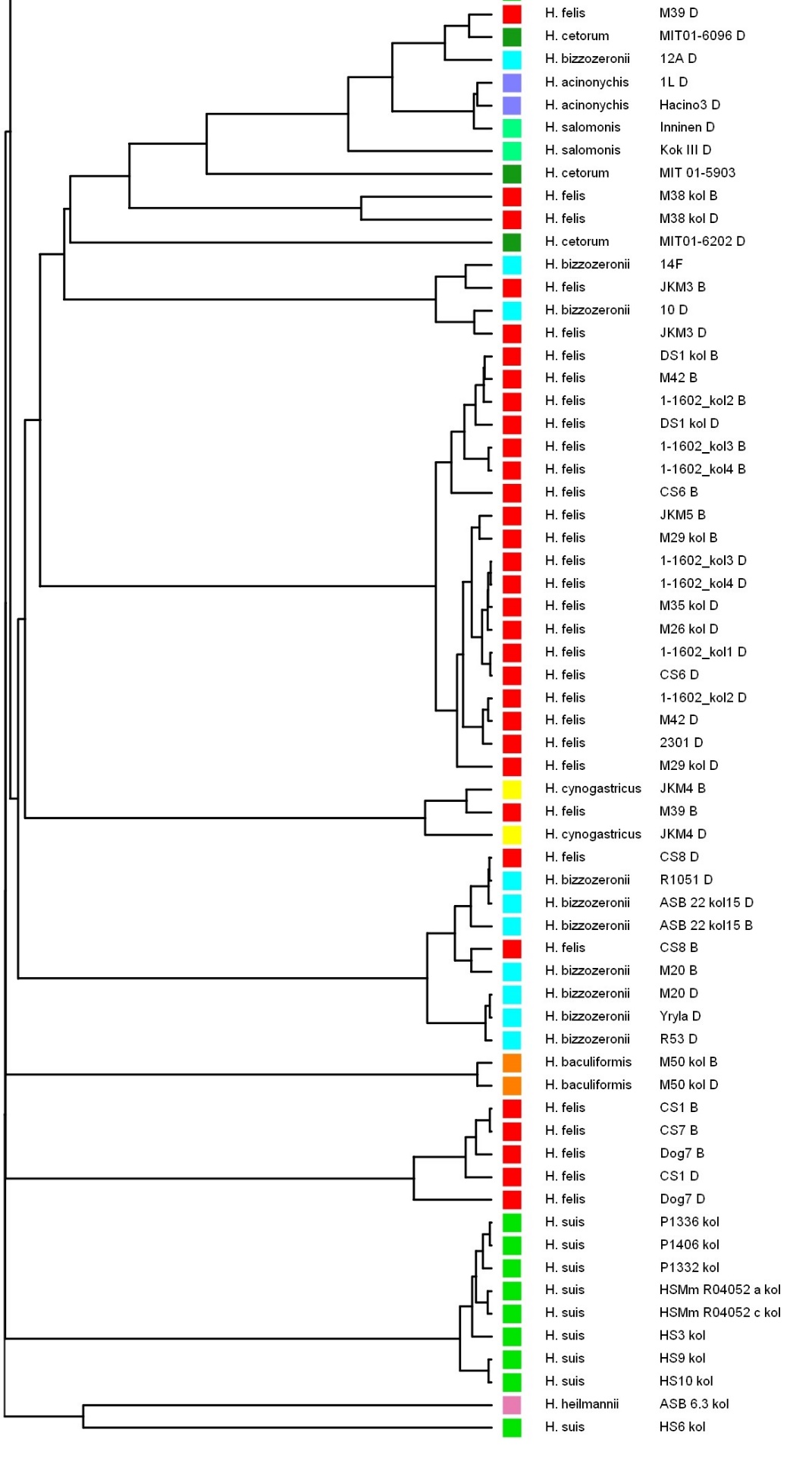


All *H. ailurogastricus* (except *H. ailurogastricus* ASB 21.1), *H. heilmannii* and *H. suis* isolates were grown under biphasic culture conditions on *Brucella* agar + *Brucella* broth. *H. ailurogastricus* ASB 21.1 was grown under dry culture conditions on *Brucella* agar.

All *H. acinonychis, H. baculiformis*, *H. bizzozeronii*, *H. cetorum*, *H. cynogastricus, H. felis*, and *H. salomonis* isolates were grown under dry (D) and/or biphasic (B) conditions on BHI agar ± BHI broth.
