# Supplementary material for: Differentiation of Gastric Helicobacter Species Using MALDI-TOF Mass Spectrometry
Source: Pathogens. 2021 Mar 18;10(3):366. doi: 10.3390/pathogens10030366 (PMC8003121; doi:10.3390/pathogens10030366)
Supplement: Supplementary file 1 [file pathogens-10-00366-s001.zip › Table S1.docx]

**Table S1.** Log scores of isolates with main spectrum profiles (MSPs) created at different timepoints

| **Species** | **20-24 individual spectra of isolate** | **D or B** | **Match** | **Log score** |
| --- | --- | --- | --- | --- |
| ***H. ailurogastricus*** | ASB7.1 kol' | B | own MSP | 2.84 |
|  |  |  | *H. ailurogastricus* ASB 7.1 kol | 2.60 |
|  | ASB 9.4 kol' | B | own MSP | 2.78 |
|  |  |  | *H. ailurogastricus* ASB 9.4 kol | 2.55 |
|  | ASB 11.2 kol | B | own MSP | 2.82 |
|  |  |  | *H. ailurogastricus* ASB 11.2 kol' | 2.57 |
|  | ASB 13.2 kol' | B | own MSP | 2.70 |
|  |  |  | *H. ailurogastricus* ASB 13.2 kol | 2.69 |
| ***H. heilmannii*** | ASB 1.4 kol' | B | own MSP | 2.77 |
|  |  |  | *H. heilmanni*i ASB 1.4 kol | 2.57 |
|  | ASB 2.1 kol' | B | own MSP | 2.82 |
|  |  |  | *H. heilmannii* ASB 2.1 kol | 2.76 |
|  | ASB 3.2 kol' | B | own MSP | 2.82 |
|  |  |  | *H. heilmannii* ASB 3.2 kol | 2.64 |
|  | ASB 6.3 kol' | B | own MSP | 2.79 |
|  |  |  | *H. heilmannii* ASB 6.3 kol | 2.74 |
|  | ASB 14.1 kol' | B | own MSP | 2.80 |
|  |  |  | *H. heilmannii* ASB 14.1 kol | 2.75 |
| ***H. suis*** | HS1 kol' | B | own MSP | 2.57 |
|  |  |  | *H. suis* HS1 kol'''' | 2.45 |
|  |  |  | *H. suis* HS1 kol | 2.42 |
|  | HS2 kol' | B | own MSP | 2.83 |
|  |  |  | *H. suis* HS2 kol'''' | 2.82 |
|  |  |  | *H. suis* HS2 kol | 2.10 |
|  | HS3 kol' | B | own MSP | 2.80 |
|  |  |  | *H. suis* HS3 kol'''' | 2.60 |
|  |  |  | *H. suis* HS3 kol | 2.11 |
|  | HS5 kol' | B | own MSP | 2.58 |
|  |  |  | *H. suis* HS5 kol | 2.42 |
|  |  |  | *H. suis* HS5 kol'''' | 1.82 |
|  | HS6 kol' | B | own MSP | 2.79 |
|  |  |  | *H. suis* HS6 kol'''' | 2.72 |
|  |  |  | *H. suis* HS6 kol'''' | 2.60 |
|  | HS9 kol' | B | own MSP | 2.83 |
|  |  |  | *H. suis* HS9 kol | 2.72 |
|  |  |  | *H. suis* HS9 kol'''' | 2.61 |
|  | HS 10 kol' | B | own MSP | 2.83 |
|  |  |  | *H. suis* HS10 kol'''' | 2.71 |
|  |  |  | *H. suis* HS10 kol | 2.66 |
|  | HSMf 331 kol' | B | own MSP | 2.82 |
|  |  |  | *H. suis* HSMf 331 kol | 2.79 |
|  | HSMf 503b kol' | B | own MSP | 2.82 |
|  |  |  | *H. suis* HSMf 503b kol | 2.34 |
|  | HSMf 504 2 kol' | B | own MSP | 2.81 |
|  |  |  | *H. suis* HSMf 504 2 kol | 2.80 |
|  | HSMf 505 2 kol' | B | own MSP | 2.47 |
|  |  |  | *H. suis* HSMf 505 2 kol | 2.23 |
|  | HSMm R02019a kol' | B | own MSP | 2.83 |
|  |  |  | *H. suis* HSMm R02019a kol | 2.39 |
|  | HSMm R02019b kol' | B | own MSP | 2.81 |
|  |  |  | *H. suis* HSMm R02019b kol | 2.13 |
|  | HSMm R04052a kol' | B | own MSP | 2.83 |
|  |  |  | *H. suis* HSMm R04052a kol | 2.66 |
|  | HSMm R04052c kol' | B | own MSP | 2.83 |
|  |  |  | *H. suis* HSMm R04052c kol | 2.54 |
|  | HSMm R07055a kol' | B | own MSP | 2.81 |
|  |  |  | *H. suis* HSMm R07055a kol | 2.69 |
|  | HSMm R07055b kol' | B | own MSP | 2.84 |
|  |  |  | *H. suis* HSMm R07055b kol | 2.62 |
|  | HSMm R07102c kol' | B | own MSP | 2.81 |
|  |  |  | *H. suis* HSMm R07102c kol | 2.43 |
|  | HSMm R08041a kol' | B | own MSP | 2.76 |
|  |  |  | *H. suis* HSMm R08041a kol | 2.61 |
|  | HSMm R08041b kol' | B | own MSP | 2.83 |
|  |  |  | *H. suis* HSMm R08041b kol | 2.63 |

D: dry cultivation; B: biphasic cultivation
