# Supplementary material for: Differentiation of Gastric Helicobacter Species Using MALDI-TOF Mass Spectrometry
Source: Pathogens. 2021 Mar 18;10(3):366. doi: 10.3390/pathogens10030366 (PMC8003121; doi:10.3390/pathogens10030366)
Supplement: Supplementary file 1 [file pathogens-10-00366-s001.zip › Table S2.docx]

**Table S2.** Logarithmic identification score matches of Brain Heart Infusion (BHI) agar with the in-house *Helicobacter* database

| **Individual spectra of** | **MSP match*** | **Log score** |
| --- | --- | --- |
| **BHI agar** | *H. salomonis* Inkinen | 2.28 |
|  | *H. cetorum* MIT 01-6096 | 2.07 |
|  | *H. bizzerozeronii* 12A | 2.05 |
|  | *H. felis* M38 | 2.01 |
|  | *H. felis* M42 | 2.01 |
|  | *H. felis* JKM3 | 1.95 |
|  | *H. felis* M39 | 1.94 |
|  | *H. bizzozeronii* 10 | 1.94 |
|  | *H. salomonis* KokIII | 1.80 |
|  | *H. acinonychis* Hacino3 | 1.69 |

MSP: main spectrum profile; *All listed isolates were grown under dry conditions using BHI agar; Green log score values: ≥ 2 acceptable for identification at species level; Orange log score values: ≥ 1.70 acceptable for identification at genus level; Red log score values < 1.70 (Bruker recommendations)
