# Supplementary material for: Differentiation of Gastric Helicobacter Species Using MALDI-TOF Mass Spectrometry
Source: Pathogens. 2021 Mar 18;10(3):366. doi: 10.3390/pathogens10030366 (PMC8003121; doi:10.3390/pathogens10030366)
Supplement: Supplementary file 1 [file pathogens-10-00366-s001.zip › Table S3.docx]

**Table S3.** Overview of spectra (*m*/*z*)[Da] showing peaks of Brain Heart Infusion (BHI) agar similar to *H. salomonis* Inkinen, *H. bizzozeronii* 12A, *H. cetorum* MIT 01-6069, and *H. felis* M38 and M42 (within a deviation range of 10 Da)

| **BHI agar** | ***H. salomonis* Inkinen** | ***H. bizzozeronii* 12A** | ***H. cetorum* MIT 01-6096** | ***H. felis***  **M38 kol** | ***H. felis***  **M42** |
| --- | --- | --- | --- | --- | --- |
| **2034** | **2036** | **2035** | **2036** | **2036** | **2035** |
|  |  |  |  | 2044 |  |
|  |  |  |  | 2046 |  |
|  | 2052 |  | 2052 | 2051 |  |
|  | 2058 | 2059 | 2060 | 2060 |  |
|  |  |  | 2064 |  | 2063 |
| **2066** | **2068** |  | **2067** | **2068** | **2067** |
|  | 2074 |  | 2073 | 2074 |  |
|  |  |  | 2082 | 2092 |  |
|  | 2108 |  | 2106 | 2107 |  |
| **2166** | **2167** |  | **2167** | **2167** |  |
|  |  |  |  | 2183 |  |
|  | 2208 | 2207 | 2207 | 2207 |  |
|  | 2224 |  |  | 2223 |  |
|  |  |  |  | 2569 |  |
|  |  |  |  | 2610 | 2609 |
|  |  |  |  | 2618 | 2618 |
|  | 2633 |  |  | 2633 |  |
|  |  |  |  | 2649 |  |
|  |  |  |  | 3343 |  |
|  |  |  |  |  | 4130 |
| **5033** |  |  |  |  | **5034** |
| **5037** | **5036** | **5035** | **5035** | **5035** |  |
|  | 5041 |  |  | 5040 | 5039 |
| **5336** | **5339** | **5339** | **5338** |  | **5338** |
|  |  |  |  |  | 5378 |
| **7550** | **7554** | **7553** | **7552** | **7552** | **7552** |
| **7557** | **7561** |  |  | **7560** | **7560** |
|  |  |  | 7570 |  |  |
| **8004** | **8010** | **8008** | **8007** | **8007** | **8007** |
|  | 8020 |  |  |  | 8021 |
|  | 8026 |  |  |  |  |
|  | 8068 |  |  |  | 8067 |
| 8157 |  |  |  |  |  |
| **15099** |  | **15102** | **15098** |  | **15106** |
|  | 15114 |  |  | 15110 |  |
| 16001 |  |  |  |  |  |
|  |  | 16013 |  |  |  |

All peaks were extracted from the mass list generated by FlexAnalysis 3.4 software after smoothing and baselining the initial spectra generated by MALDI-TOF MS (Bruker Daltonics).
