# Supplementary material for: Differentiation of Gastric Helicobacter Species Using MALDI-TOF Mass Spectrometry
Source: Pathogens. 2021 Mar 18;10(3):366. doi: 10.3390/pathogens10030366 (PMC8003121; doi:10.3390/pathogens10030366)
Supplement: Supplementary file 1 [file pathogens-10-00366-s001.zip › Table S4.docx]

**Table S4.** *Helicobacter* isolates used in the current study to construct the in-house *Helicobacter* main spectrum profile (MSP) database

| ***Helicobacter* species** | **Isolate** | **Host of isolation** | **Culture conditions** | ***In vitro* passage** | **Accession number*** |
| --- | --- | --- | --- | --- | --- |
| *H. acinonychis* | 1L  90-624  Hacino3  SB-1 | Sumatrian tiger  cheetah  lion  Bengal tiger | dry + biphasic, BHI agar ± broth, pH 7, 37°C, microaerobic  biphasic, BHI agar + broth, pH 7, 37°C, microaerobic  dry + biphasic, BHI agar ± broth, pH 7, 37°C, microaerobic  dry, BHI agar, pH 7, 37°C, microaerobic | unknown  unknown  unknown  unknown | FZLX00000000^b^  FZMD00000000^b^  FZMC00000000^b^  FZLV00000000^b^ |
| *H. ailurogastricus* | ASB 7.1^T^  ASB 9.4  ASB 11.2  ASB 13.2  ASB 21.1  ASB 23 | cat  cat  cat  cat  cat  cat | biphasic, *Brucella* agar + broth, pH 5, 37°C, microaerobic  biphasic, *Brucella* agar + broth, pH 5, 37°C, microaerobic  biphasic, *Brucella* agar + broth, pH 5, 37°C, microaerobic  biphasic, *Brucella* agar + broth, pH 5, 37°C, microaerobic  dry, *Brucella* agar, pH 5, 37°C, microaerobic  biphasic, *Brucella* agar + broth, pH 5, 37°C, microaerobic | unknown  unknown  unknown  unknown  unknown  11 | CDMG00000000  CDMN00000000  CDML00000000  CDMH00000000  FZLU00000000^b^  FZMH00000000^b^ |
| *H. baculiformis* | M50^T^ | cat | dry + biphasic, BHI agar ± broth, pH 7, 37°C, microaerobic | unknown | FZMF00000000^b^ |
| *H. bizzozeronii* | 10  12A  14F  ASB 22 kol 15  Heydar  M20  R53  R1051  Yryla | dog  dog  dog  cat  dog  dog  human  dog  dog | dry, BHI agar, pH 7, 37°C, microaerobic  dry, BHI agar, pH 7, 37°C, microaerobic  dry, BHI agar, pH 7, 37°C, microaerobic  dry + biphasic, BHI agar ± broth, pH 7, 37°C, microaerobic  biphasic, BHI agar + broth, pH 7, 37°C, microaerobic  dry + biphasic, BHI agar ± broth, pH 7, 37°C, microaerobic  dry, BHI agar, pH 7, 37°C, microaerobic  dry, BHI agar, pH 7, 37°C, microaerobic  dry, BHI agar, pH 7, 37°C, microaerobic | unknown  unknown  unknown  unknown  unknown  unknown  unknown  unknown  unknown | FZEH00000000^b^  FZMK00000000^b^  FZLJ00000000^b^  FZKO00000000^b^  FZLB00000000^b^  FZMY00000000^b^  FZKR00000000^b^ |
| *H. cetorum* | MIT 01-5903  MIT 01-6202  MIT 01-6096 | Pacific white sided dolphin  Atlantic bottle nose dolphin  Atlantic bottle nose dolphin | dry, BHI agar, pH 7, 37°C, microaerobic  dry, BHI agar, pH 7, 37°C, microaerobic  dry, BHI agar, pH 7, 37°C, microaerobic | unknown  unknown  unknown | FZMR00000000^b^  FZMU00000000^b^ |
| *H. cynogastricus* | JKM4^T^ | dog | dry + biphasic, BHI agar ± broth, pH 7, 37°C, microaerobic | unknown | FZMQ00000000^b^ |
| *H. felis* | 1-1602 kol1  1-1602 kol 2  1-1602 kol 3  1-1602 kol 4  2301  16937  CS1^T^  CS6  CS7  CS8  Dog7  DS1  JKM3  JKM5  M26  M29  M35  M38  M39  M42 | dog  dog  dog  dog  dog  dog  cat  cat  cat  cat  dog  dog  dog  dog  dog  dog  dog  dog  dog  dog | dry, BHI agar, pH 7, 37°C, microaerobic  dry + biphasic, BHI agar ± broth, pH 7, 37°C, microaerobic  dry + biphasic, BHI agar + broth, pH 7, 37°C, microaerobic  dry + biphasic, BHI agar + broth, pH 7, 37°C, microaerobic  dry, BHI agar, pH 7, 37°C, microaerobic  biphasic, BHI agar + broth, pH 7, 37°C, microaerobic  dry + biphasic, BHI agar ± broth, pH 7, 37°C, microaerobic  dry + biphasic, BHI agar + broth, pH 7, 37°C, microaerobic  biphasic, BHI agar + broth, pH 7, 37°C, microaerobic  dry + biphasic, BHI agar ± broth, pH 7, 37°C, microaerobic  dry + biphasic, BHI agar ± broth, pH 7, 37°C, microaerobic  dry + biphasic, BHI agar ± broth, pH 7, 37°C, microaerobic  dry + biphasic, BHI agar ± broth, pH 7, 37°C, microaerobic  biphasic, BHI agar + broth, pH 7, 37°C, microaerobic  dry, BHI agar, pH 7, 37°C, microaerobic  dry + biphasic, BHI agar ± broth, pH 7, 37°C, microaerobic  dry, BHI agar, pH 7, 37°C, microaerobic  dry + biphasic, BHI agar ± broth, pH 7, 37°C, microaerobic  dry + biphasic, BHI agar ± broth, pH 7, 37°C, microaerobic  dry + biphasic, BHI agar ± broth, pH 7, 37°C, microaerobic | unknown  unknown  unknown  unknown  unknown  unknown  unknown  unknown  unknown  unknown  unknown  unknown  unknown  unknown  unknown  unknown  unknown  unknown  unknown  unknown | FZLC00000000^b^  FZLH00000000^b^  FZLL00000000^b^  FZKQ00000000^b^  FZKU00000000.1  NC_014810  FZKM00000000^b^  FZKX00000000^b^  FZKG00000000^b^  FZLG000000000^b^  FZNI00000000^b^  FZKW00000000^b^  FZKZ00000000^b^  FZKS00000000^b^  FZLF00000000^b^  FZKK00000000^b^  FZKF00000000^b^  FZKP00000000^b^  FZLA00000000^b^ |
| *H. heilmannii* | ASB 1.4^T^  ASB 2.1  ASB 3.2  ASB 6.3  ASB 14.1  ASB 19.4  ASB 20.2 | cat  cat  cat  cat  cat  cat  cat | biphasic, *Brucella* agar + broth, pH 5, 37°C, microaerobic  biphasic, *Brucella* agar + broth, pH 5, 37°C, microaerobic  biphasic, *Brucella* agar + broth, pH 5, 37°C, microaerobic  biphasic, *Brucella* agar + broth, pH 5, 37°C, microaerobic  biphasic, *Brucella* agar + broth, pH 5, 37°C, microaerobic  biphasic, *Brucella* agar + broth, pH 5, 37°C, microaerobic  biphasic, *Brucella* agar + broth, pH 5, 37°C, microaerobic | unknown  15  13  19  5  15  20 | CDMK00000000  CDMP00000000  CDMJ00000000  CDMM00000000  CDMI00000000  FZMG00000000^b^  FZME00000000^b^ |
| *H. salomonis* | Alma0595  Elvira II  Inkinen^T^  KokIII  M45  MINI13  R1053 | dog  dog  dog  dog  dog  dog  dog | dry + biphasic, BHI agar ± broth, pH 7, 37°C, microaerobic  dry + biphasic, BHI agar ± broth, pH 7, 37°C, microaerobic  dry + biphasic, BHI agar ± broth, pH 7, 37°C, microaerobic  dry + biphasic, BHI agar ± broth, pH 7, 37°C, microaerobic  dry, BHI agar, pH 7, 37°C, microaerobic  dry, BHI agar, pH 7, 37°C, microaerobic  dry, BHI agar, pH 7, 37°C, microaerobic | unknown  unknown  unknown  unknown  unknown  unknown  unknown | FZMB00000000^b^  FZMA00000000^b^  FZLZ00000000^b^  FZLY00000000^b^  OANQ00000000^b^ |
| *H. suis* | HS1^T^  HS2  HS3  HS4  HS5  HS6  HS7  HS8  HS9  HS10  P13/04  P13/24  P13/26  P13/28  P13/32  P13/35  P13/36  P14/06  P14/09  P14/10  HSMf 331  HSMf 503b  HSMf 504/1  HSMf 504/2  HSMf 505/1  HSMf 505/2  HSMm R02019a  HSMm R02019b  HSMm R04052a  HSMm R04052c  HSMm R07055a  HSMm R07055b  HSMm R07102c  HSMm R08041a  HSMm R08041b | pig  pig  pig  pig  pig  pig  pig  pig  pig  pig  pig  pig  pig  pig  pig  pig  pig  pig  pig  pig  *Macaca fascicularis*  *Macaca fascicularis*  *Macaca fascicularis*  *Macaca fascicularis*  *Macaca fascicularis*  *Macaca fascicularis*  *Macaca mulatta*  *Macaca mulatta*  *Macaca mulatta*  *Macaca mulatta*  *Macaca mulatta*  *Macaca mulatta*  *Macaca mulatta*  *Macaca mulatta*  *Macaca mulatta* | biphasic, *Brucella* agar + broth, pH 5, 37°C, microaerobic  biphasic, *Brucella* agar + broth, pH 5, 37°C, microaerobic  biphasic, *Brucella* agar + broth, pH 5, 37°C, microaerobic  biphasic, *Brucella* agar + broth, pH 5, 37°C, microaerobic  biphasic, *Brucella* agar + broth, pH 5, 37°C, microaerobic  biphasic, *Brucella* agar + broth, pH 5, 37°C, microaerobic  biphasic, *Brucella* agar + broth, pH 5, 37°C, microaerobic  biphasic, *Brucella* agar + broth, pH 5, 37°C, microaerobic  biphasic, *Brucella* agar + broth, pH 5, 37°C, microaerobic  biphasic, *Brucella* agar + broth, pH 5, 37°C, microaerobic  biphasic, *Brucella* agar + broth, pH 5, 37°C, microaerobic  biphasic, *Brucella* agar + broth, pH 5, 37°C, microaerobic  biphasic, *Brucella* agar + broth, pH 5, 37°C, microaerobic  biphasic, *Brucella* agar + broth, pH 5, 37°C, microaerobic  biphasic, *Brucella* agar + broth, pH 5, 37°C, microaerobic  biphasic, *Brucella* agar + broth, pH 5, 37°C, microaerobic  biphasic, *Brucella* agar + broth, pH 5, 37°C, microaerobic  biphasic, *Brucella* agar + broth, pH 5, 37°C, microaerobic  biphasic, *Brucella* agar + broth, pH 5, 37°C, microaerobic  biphasic, *Brucella* agar + broth, pH 5, 37°C, microaerobic  biphasic, *Brucella* agar + broth, pH 5, 37°C, microaerobic  biphasic, *Brucella* agar + broth, pH 5, 37°C, microaerobic  biphasic, *Brucella* agar + broth, pH 5, 37°C, microaerobic  biphasic, *Brucella* agar + broth, pH 5, 37°C, microaerobic  biphasic, *Brucella* agar + broth, pH 5, 37°C, microaerobic  biphasic, *Brucella* agar + broth, pH 5, 37°C, microaerobic  biphasic, *Brucella* agar + broth, pH 5, 37°C, microaerobic  biphasic, *Brucella* agar + broth, pH 5, 37°C, microaerobic  biphasic, *Brucella* agar + broth, pH 5, 37°C, microaerobic  biphasic, *Brucella* agar + broth, pH 5, 37°C, microaerobic  biphasic, *Brucella* agar + broth, pH 5, 37°C, microaerobic  biphasic, *Brucella* agar + broth, pH 5, 37°C, microaerobic  biphasic, *Brucella* agar + broth, pH 5, 37°C, microaerobic  biphasic, *Brucella* agar + broth, pH 5, 37°C, microaerobic  biphasic, *Brucella* agar + broth, pH 5, 37°C, microaerobic | 26  24  19  28  21  24  22  27  26  17  28  23  25  21  25  27  25  22  17  21  6  6  7  8  9  7  8  8  6  8  8  7  8  8  8 | ADGY00000000  FZLI00000000^b^  FZKT00000000^b^  FZKI00000000^b^  FZKN00000000^b^  FZLD00000000^b^  FZKH00000000^b^  FZKJ00000000^b^  FZLE00000000^b^  FZKV00000000^b^  GCA_902312335.1  GCA_902312325.1  GCA_902312345.1  GCA_902196125.1  GCA_902196095.1  GCA_902196115.1    GCA_902196135.1    GCA_902196105.1  GCA_902196145.1  GCA_902196155.1 |

BHI: Brain Heart Infusion

microaerobic: 85% N_2_, 10% CO_2_ and 5% O_2_

^T^type strain

^b^EMBL accession numbers can be found via Bioproject record number PRJEB21369

*Genome accession number; only isolates with a genome accession number were included in the phylogenetic analysis (Figure S5)
